# Moving beyond global scores: the added value of clinical dimensions for assessing HRQoL in mental disorders

**DOI:** 10.1007/s11136-025-04099-3

**Published:** 2026-01-09

**Authors:** Yeujin Ki, Andrew Athan McAleavey, Tron Anders Moger, Christian Moltu

**Affiliations:** 1 Department of Research and Innovation, Helse Førde, Svanehaugvegen 2, 6812 Førde, Norway; 2 Department of Psychiatry, Helse Førde, Svanehaugvegen 2, Førde, 6812 Norway; 3 Department of Health and Caring Sciences, Western Norway University of Applied Science, Svanehaugvegen 1, Førde, 6812 Norway; 4 Department of Health Management and Health Economics, University of Oslo, Problemveien 11, 0313 Oslo, Norway

**Keywords:** Quality of life (QoL), Health-related quality of life (HRQoL), Routine outcome monitoring, Clinical feedback systems, Specialist mental healthcare

## Abstract

**Purpose:**

Health-Related Quality of Life (HRQoL) is an important outcome in health services research, but the suitability of generic HRQoL instrument for mental health populations is debated. This study investigates how clinical mental health dimensions (Norse Feedback, NF) supplement the generic HRQoL instruments (EuroQol five-dimensions five-level, EQ-5D-5 L) to explain their subjective well-being, defined by self-rated health (EuroQol Visual Analogue Scale, EQ-VAS) and life satisfaction.

**Methods:**

In a cross-sectional study, 307 adults in specialized mental health treatment in Norway completed the EQ-5D-5 L and the modified NF. We compared a baseline regression model using only EQ-5D-5 L dimensions with a comprehensive model using factors derived from an Exploratory Factor Analysis of all 33 combined dimensions: 5 from EQ-5D-5 L and 28 from NF.

**Results:**

Factor analysis yielded five factors, two of which (‘Behavioral problems/externalizing’ and ‘Social/life stability’) consisted exclusively of clinical dimensions not captured by the EQ-5D-5L. A comprehensive model using these factor scores significantly improved explanatory power over a baseline EQ-5D-5L model. The adjusted R² increased by 8.4% points for EQ-VAS (from 23.5 to 31.9%) and by 17.3% points for life satisfaction (from 41.1% to 58.4%). The dominant ‘Psychological distress’ factor was key to this improvement, integrating the single EQ-5D-5L ‘Anxiety/depression’ dimension with eleven clinical dimensions like Internal avoidance and Hopelessness.

**Conclusion:**

While the EQ-5D-5 L is valuable, its assessment of well-being in this population is significantly enhanced by incorporating some clinical dimensions. Particularly, mental health-specific dimensions such as Internal avoidance, Intrusive memories, Self-contempt and Hopelessness played crucial roles to understand the gap between generic HRQoL and their sense of overall well-being.

## Introduction

 Quality of Life (QoL) is a fundamental concept in understanding human well-being. The World Health Organization (WHO) defines QoL as an “individuals’ perception of their position in life in the context of the culture and value systems in which they live and in relation to their goals, expectations, standards and concerns“ [1]. This definition underscores that QoL is inherently subjective, multidimensional, and culturally situated, encompassing aspects like physical health, psychological state, social relationships, and personal beliefs within one’s environment [1]. Within health services research, especially in health economics, a key focus is often on Health-Related Quality of Life (HRQoL). According to the U.S. Centers for Disease Control and Prevention (CDC), HRQoL relates to “an individual’s or a group’s perceived physical and mental health over time” [2]. This definition emphasizes the subjective perception of both physical and mental health aspects that affects QoL.

While various definitions exist [3], in health economics, HRQoL is frequently operationalized through utility score, representing ‘values assigned to various health states’ on a scale from 0 (death) to 1(full health) [4]. These scores are typically derived using standardized HRQoL instruments. Among these, EuroQoL’s five-dimensional questionnaire (EQ-5D) has achieved a prominent position. This prominence is evident in pharmacoeconomic guidelines globally, where 29 out of 34 National Health Technology Assessment guidelines incorporating EQ-5D, with 15 specifically designating it as their preferred instrument [5]. This widespread adoption has been driven by influential key agencies in the UK [6] and Netherlands [7], which have officially adopted EQ-5D in their reference cases [8]. In Norway, expert groups also recommend EQ-5D-5L for ‘describing and valuing various health conditions (HRQoL)’ [9]. This widespread adoption as a de facto standard, however, raises a critical question about its suitability for all populations, particularly those with mental health conditions. For instance, a study conducted in the UK found EQ-5D had significant limitations in capturing the health-related impacts of certain conditions, including mental health [10]. Similarly, a study comparing the EQ-5D with the QoL instrument developed by the World Health Organization (WHOQoL) has demonstrated that the EQ-5D is less sensitive to changes in social and psychological well-being in particular and concludes that it seems “less appropriate to use it as a core measure in economic health evaluation in the field of psychiatry” [11]. Given its generic nature, there is a crucial need to understand which aspects of well-being may be overlooked when relying predominantly on this single measure.

While EQ-5D’s performance for depression and anxiety is generally regarded as satisfactory [12, 13], some studies have suggested limitations even in these areas [14, 15]. Additionally, it has also been observed that it may not be sufficiently responsive in measuring changes in HRQoL in other mental conditions including bipolar disorder, schizophrenia spectrum and other psychotic disorders [16–18]. These recognized challenges have prompted efforts to enhance EQ-5D, such as exploring complementary bolt-on dimensions for diverse conditions [19]. For instance, studies by Chen and Olsen [20, 21] demonstrated that adding psycho-social dimensions (Vitality, Sleep, Social Relationships, Community Connectedness) were significantly associated with improved performance in explaining life satisfaction and self-rated health (VAS-Health Status, EQ-VAS). Their findings also revealed a more complex picture by showing that the key factors influencing a sense of overall well-being differ between the general population and those with depressive conditions [21].

Building on this existing knowledge, our study is an attempt to identify these assessment gaps. We incorporate a broad set of 28 clinical mental health dimensions, which were designed to be transdiagnostic and applicable to patient with a wide array of common clinical concerns (e.g., Sleep, Cognitive problems, and Hopelessness). Our objective is twofold: First, we examine correlations between EQ-5D-5 L dimensions and the 28 clinical dimensions to explore their relationships. Second, we test whether clinical mental health dimensions predict participants’ general sense of health (EQ-VAS) and life satisfaction (LS) beyond the generic EQ-5D-5 L. To achieve this, we compare a baseline regression model using only EQ-5D-5 L dimensions with a comprehensive model using factors derived from an Exploratory Factor Analysis (EFA) of all 33 combined dimensions. Through these analyses, we aim to identify mental health dimensions that could complement EQ-5D-5 L, thereby providing a more comprehensive framework for mental health assessment.

## Method and materials

### Study design

This paper reports on a quantitative investigation within a larger research project that explores the HRQoL of individuals with mental disorders. A corresponding qualitative study was conducted with a subset of participants, and its findings are published elsewhere [22].

### Participants

Inclusion criteria were individuals > 18 years old, currently in active inpatient or outpatient treatment in specialized mental health services in Førde Hospital Trust, a regional health enterprise in Norway. We applied no diagnostic exclusion criteria to capture diverse patient experiences. However, the requirement to provide electronic informed consent ensured that all participants had the necessary capacity to understand the study and agree to participate.

### Measures

Participants completed EQ-5D-5 L and modified NF between June 2023 and October 2024.

The EQ-5D-5 L, a widely used HRQoL instrument, consists of two components: a descriptive system and a visual analogue scale (EQ-VAS) [23]. The descriptive system measures five dimensions (Mobility, Self-care, Usual activities, Pain/discomfort, and Anxiety/depression) on a 5-point Likert scale which are converted to utility scores ranging from 0 (death) to 1 (perfect health) [8]. This structure, with four of its five dimensions focused primarily on physical health and general functioning, highlights the instrument’s generic nature. This forms the basis for our investigation into which additional, mental health-specific concepts are needed to supplement its assessment of well-being in mental health population. While the descriptive system assesses five specific health dimensions, the EQ-VAS health status captures the respondent’s overall self-rated health on a vertical scale ranging from 0 (‘worst imaginable health state’) to 100 (‘best imaginable health state’). We used the EQ-VAS score as one of the variables for participants’ sense of overall well-being, as it has been reported to encompass a broader construct than the EQ-5D-5 L descriptive system index (utility score) [24].

NF is a clinical self-report measure used and researched at scale in Norway [25–27] and USA [28–30] which assesses broader aspects of mental health across 28 dimensions, each rated on a 7-point scale [25]. While the clinically used NF employs trigger questions to filter out subsequent items, the modified version used in this study included all items without such filtering, excluding eight less relevant items - therapeutic alliance related items (e.g., agreement on treatment goals, therapeutic relationship) and meta-reflective items about the assessment process itself (e.g., ‘I feel that completing these questions can be important to my treatment’). For better understanding, dimension names were modified to be more specific. For example, items under “Social determinants items” such as “I have enough money for my needs” were reclassified under more concrete dimensions like “Financial security.” Complete details of original and modified dimension names are available in Supplement 5.

We utilized the life satisfaction item in NF (“Overall, how satisfied are you with your life right now? [0–10]”) as another measure of participants’ overall well-being, along with the EQ-VAS health status. Although the NF labels this item as ‘Quality of Life’, the question explicitly asks about life satisfaction. Therefore, in this study, we refer to this item as Life Satisfaction (LS) to more accurately reflect the construct being measured. 

### Data analysis

#### Data preparation and preliminary analysis

Descriptive statistics were calculated for all variables. Addressing our first objective to explore the relationships between measures, Pearson correlations assessed relationships between the EQ-5D-5 L, NF dimensions, and the main outcomes (EQ-VAS and LS). The strength of correlations was interpreted using Cohen’s criteria [31].

To facilitate the comparison of effects in the regression models, all variables were standardized to z-scores prior to the main analysis.

#### Model development and exploratory factor analysis (EFA)

To address our second objective, this analysis aims to identify which underlying clinical constructs supplement the generic EQ-5D-5 L in explaining patient well-being, and to quantify the additional explanatory power gained by the broader assessment using these dimensions. To achieve this, we compared two multivariable linear regression models for each of our outcomes.

The first model, the ‘baseline EQ-5D-5L model’ served to establish the explanatory power of the generic HRQoL instrument alone. This model included the five dimensions from the EQ-5D-5 L (Mobility, Self-care, Usual activities, Pain/discomfort, and Anxiety/depression) as predictor variables.

The second model, the ‘comprehensive model’ was designed to capture the comprehensive information within all 33 available dimensions (5 from EQ-5D-5 L and 28 from NF). Given the substantial intercorrelations among these dimensions, we performed an EFA as a preliminary dimension reduction step to identify the underlying latent constructs across the EQ-5D-5 L and NF. The resulting factor scores represent a model-simplified structure parsimoniously combining the two instruments’ items. We chose Principal axis factoring to focus on latent constructs, and a Promax rotation to allow for realistic correlations between these constructs. The optimal number of factors to extract was determined using parallel analysis, examination of the scree plot, and factor loading matrix interpretation. Factor scores based on the selected model were generated and used as predictors for the comprehensive model.

#### Model comparison

Finally, the baseline EQ-5D-5 L model and the comprehensive models were compared to evaluate the added value of the broader assessment. The practical improvement in explanatory power was quantified by examining the change in adjusted R^2^. The Akaike Information Criterion (AIC) was used for a formal statistical comparison of relative model fit. The model with the lower AIC value was considered to represent a better balance between model fit and parsimony. Data analysis was performed using R ver. 4.1.0.

## Results

### Participant characteristics

A total of 5,806 individuals currently receiving treatment in specialized mental health services were approached with an open invitation for participation, of whom 307 (5.3%) agreed to participate and completed the questionnaires. The study population’s mean EQ-5D-5 L utility score was 0.61 (SD = 0.2), which was lower than the Norwegian general population norm of 0.80 (SD = 0.2) [32]. Similarly, our participants’ mean EQ-VAS score of 54.7 (SD = 21.3) fell well below the general population average of 77.9 (SD = 18.3) [32]. These values in our sample more closely resemble those reported for individuals with depression in primary care (utility scores: 0.60–0.70; EQ-VAS scores: 53–67) [33] and for psychiatric outpatients in specialist mental healthcare (utility scores: 0.46; EQ-VAS: 47.5) [34]. The mean age of the study population was 38.3 years (SD = 13.5), and 213 (69.4%) were female. Table 1 presents the detailed participant characteristics.

**Table 1:**
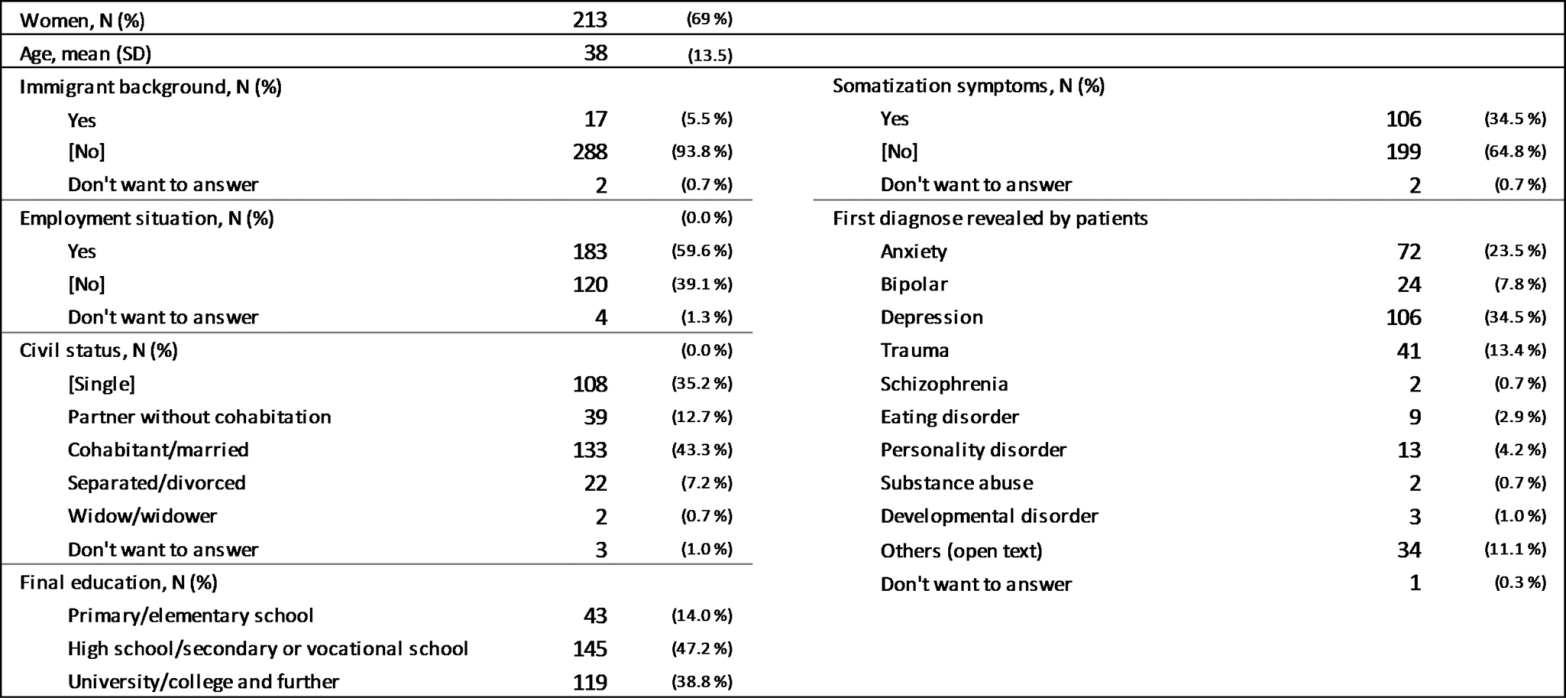
Participant characteristics (*N* = 307)

### Correlation between EQ-5D-5 L and NF dimensions with sense of overall well-being

As a preliminary step, a full correlation analysis was performed between all measured dimensions. The correlations among dimensions within the EQ-5D-5 L and the NF respectively were also examined as part of this.

**Fig. 1:**
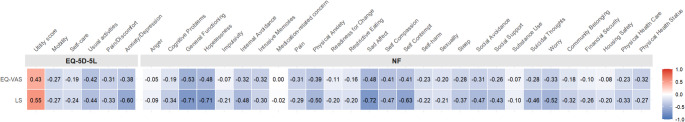
Pearson correlations of EQ-5D-5 L and NF dimensions with well-being outcomes (see online version for color figure)

Figure 1 presents the relationship between all dimensions and participants’ overall well-being. EQ-5D-5 L utility score showed moderate correlations with EQ-VAS (0.43) and relatively stronger correlations with LS (0.55). Among the dimensions, Usual activities (− 0.42) had the strongest association with EQ-VAS, while Anxiety/depression (− 0.60) was most strongly correlated with LS. Mobility and Self-care dimensions showed relatively weaker correlations with both well-being outcomes (− 0.27 ~ − 0.19). NF’s clinical mental health dimensions had relatively stronger associations with EQ-VAS and LS than EQ-5D-5 L dimensions. For instance, EQ-VAS showed strong correlations with General functioning (− 0.53) while demonstrating moderate correlations with Sad affect (− 0.48), Hopelessness (− 0.48) and Self-contempt (− 0.41). Similarly, LS demonstrated strong correlations with General functioning (− 0.71), Sad affect (− 0.72), Hopelessness (− 0.71), and Self-contempt (− 0.63).

**Fig. 2:**
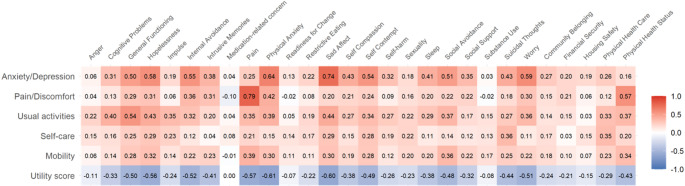
Pearson correlation between EQ-5D-5 L and NF dimensions (see online version for color figure)

Finally, Fig. 2 presents the relationship between EQ-5D-5 L and clinical mental health dimensions of NF. It revealed expected relationships between conceptually similar dimensions. As expected, NF’s Pain dimension showed the strongest correlation with EQ-5D-5 L Pain/discomfort dimension (0.79), while NF’s Sad affect (0.74) and Physical anxiety (0.64) showed strong correlations with EQ-5D-5 L Anxiety/depression dimension. These strong correlations demonstrate conceptual overlap between related dimensions of the two measures.

Meanwhile, it is noteworthy that other aspects of mental health dimensions - such as Hopelessness (0.58), Internal avoidance (0.55), Self-contempt (0.54), Social avoidance (0.51), and Worry (0.59) - were predominantly associated with a single EQ-5D-5 L dimension, Anxiety/depression. The correlations in the Usual activities dimension showed moderate associations across multiple NF domains, particularly with General functioning (0.54), indicating its broader relationship with overall functional status. Self-care and Mobility dimensions generally showed weaker correlations with NF domains, with most correlations falling below 0.40, suggesting these physical health dimensions capture distinct aspects from the primarily mental health-focused NF domains.

### Predicting sense of overall well-being

The high correlations observed among the variables suggested that the scales might share common underlying factors. Accordingly, we conducted EFA on all scores generated by the NF and EQ-5D-5 L dimensions together to investigate the underlying latent structure and inform subsequent model building. Analysis of the scree plot and simulated data using parallel analysis resulted in a five-factor solution. Analysis results and factor loadings are presented in Supplement 2 and 3.

The selected five-factor structure included highly recognizable and interpretable factors. Based on our assessment of the items that loaded strongly onto each factor, we assigned the following descriptive labels ‘Psychological distress’, ‘Pain/physical health’, ‘Functioning/self-management’, ‘Behavioral problems/externalizing’ and ‘Social/life stability’.

A robust ‘Psychological distress’ factor emerged, combining the EQ-5D-5L ‘Anxiety/depression’ dimension with items from the NF’s scales touching on common symptoms of negative affect: physical anxiety, worry, sad affect, and intrusive memories, among others. A ‘Pain & physical health’ factor also emerged, which grouped physical health items from both instruments. Interestingly, for this population, this factor combined the theoretically distinct EQ-5D-5L dimensions of ‘Pain/discomfort’ and ‘Mobility’. Other factors highlighted the unique contributions of each instrument; the ‘Functioning & self-management’ factor was primarily defined by the EQ-5D-5L ‘Usual activities’ and ‘Self-care’ items, while the ‘Behavioral problems/externalizing’ and ‘Social/life stability’ factors were composed exclusively of clinical and social dimensions from the NF. Notably, the ‘Functioning & self-management’ and ‘Behavioral problems/externalizing’ factors were defined by a small number of items, suggesting the estimates should be interpreted with caution. Finally, several clinically important dimensions, including ‘Restrictive eating’ and ‘Sexuality’, did not load strongly onto any of the five factors and were thus excluded from further analysis.

To quantify the incremental predictive value of broader clinical dimensions, we compared the performance of the comprehensive model against the baseline EQ-5D-5 L model using the adjusted R^2^.

**Table 2:** Regression results predicting EQ-VAS and LS scores

		EQ-VAS		LS	
		Coefficient	*p*-value	Coefficient	*p*-value
Baseline EQ-5D-5 L model	Mobility	− 0.04	0.54	0.04	0.52
	Self-care	0.03	0.61	− 0.01	0.90
	Usual activities	− 0.29	< 0.01*	− 0.23	< 0.01*
	Pain/discomfort	− 0.12	0.05	− 0.09	0.07
	Anxiety/depression	− 0.22	< 0.01*	− 0.50	< 0.01*
	Adjusted R^2^	0.235		0.411	
	AIC	797.1		716.5	
Comprehensive (EQ-5D-5 L + NF) model	Factor 1: Psychological distress	− 0.36	< 0.01*	− 0.52	< 0.01*
	Factor 2: Pain/ physical health	− 0.22	< 0.01*	− 0.09	0.04*
	Factor 3: Functioning/ self-management	− 0.24	< 0.01*	− 0.34	< 0.01*
	Factor 4: Behavioral problems/externalizing	0.21	< 0.01*	0.23	< 0.01*
	Factor 5: Social/ life stability	− 0.03	0.67	− 0.15	< 0.01*
	Adjusted R^2^	0.319		0.584	
	AIC	761.3		610.0	

^*^(p < 0.05)

For EQ-VAS scores, the baseline EQ-5D-5 L model explained 23.5% of the variance, while the comprehensive model achieved 31.9%, representing an 8.4% points (pp) increase. The improvement was more substantial for LS scores, where the baseline model’s explanatory power increased from 41.1 to 58.4% in the comprehensive model, yielding a 17.3 pp again.

Model comparison using the AIC further confirmed the superiority of the comprehensive model. Lower AIC values were observed for both EQ-VAS and LS, indicating that the comprehensive model achieved a better balance between model fit and parsimony. Detailed regression results for both models are presented in Table 2.

### Sensitivity analyses

Sensitivity analyses were conducted using sex and age as moderators to compare model robustness. The results indicated that the baseline EQ-5D-5 L model exhibited several significant interactions with both sex and age. In contrast, no statistically significant interactions were observed for the comprehensive model with either moderator. These findings suggest that the comprehensive model is more robust to demographic moderators than the baseline EQ-5D-5 L model. The full results of the sensitivity analyses are presented in the Supplement 4.

## Discussion

This study aimed to examine correlations between EQ-5D-5 L dimensions and the 28 clinical NF dimensions, and to test whether these specific clinical dimensions predict participants’ general sense of health (EQ-VAS) and life satisfaction (LS) above and beyond the generic EQ-5D-5 L. Results showed that a model incorporating more detailed clinical self-report constructs explained a considerably greater proportion of the variance in both EQ-VAS and LS compared to the baseline model. Specifically, the inclusion of clinical factors increased the adjusted R^2^ by 8.4 pp for EQ-VAS (from 23.5 to 31.9%) and 17.3 pp for LS (from 41.1 to 58.4%). The superiority of the comprehensive model was also confirmed by lower AIC values for both well-being outcomes.

The results of the EFA help to clarify the source of this increased explanatory power. First, the analysis identified factors that represent dimensions of patient experience not directly captured by the generic EQ-5D-5L instrument. The ‘Behavioral problems/externalizing’ and ‘Social/life stability’ factors were composed exclusively of dimensions from the NF instrument, such as anger, impulsivity, social support, and housing safety. This finding suggests that the EQ-5D-5 L may be missing key psycho-social concepts that are important determinants of quality of life for individuals with mental disorders.

This finding aligns with previous research investigating the incremental value of supplementary dimensions to the EQ-5D. Studies focused on developing and testing ‘bolt-on’ items have consistently demonstrated that dimensions beyond the core EQ-5D —for instance, relationships, satisfaction, and vitality—significantly increase the model’s ability to explain variance in self-rated health (VAS) [35]. Our results corroborate this conclusion in a specialized mental health context, demonstrating that granular clinical and psycho-social dimensions offer substantial incremental value.

Second, the improvement in the model’s predictive power may stem from the model’s ability to capture not just symptoms, but also the process of suffering. This is best illustrated by the ‘Psychological distress’ factor, which was the strongest predictor of both well-being outcomes. In this factor, the ‘Anxiety/depression’ dimension from the EQ-5D-5 L co-loaded with eleven clinical dimensions from the NF, such as Internal avoidance, Intrusive memories and Self-contempt. While the EQ-5D-5 L dimension focuses on the outcome of distress (e.g., “I am anxious or depressed”), the ‘Psychological distress’ factor additionally assesses the maladaptive process of struggling against it. For instance, items such as “I spend a lot of energy not to think about things that hurt (Internal avoidance)”, “I can’t make my worries stop, I have been overwhelmed by my worries (worry)”, or “I adjust my life to control my terrifying memories (Intrusive memories)” provide much more specific and proximal causes or instances of anxiety and distress. This focus on process is supported by contemporary findings, such as those demonstrating the link between maladaptive coping and QoL impairment [36]. Specifically, research shows that when patients attempt to suppress or avoid their distress, this behavior exacerbates the resulting functional decline and subsequent impairment of QoL [36]. This mechanism - where the continuous effort to avoid painful thoughts and memories actively narrows one’s life activities-may itself be a primary cause of functional decline and reduced quality of life. This suggests that while the EQ-5D-5 L can signal the presence of distress, the more detailed clinical items provide critical specificity about the nature of that suffering.

Furthermore, the clinical dimensions included items that encompass more temporally broad states or future expectations. For instance, EQ-5D-5 L’s Usual activities and Self-care dimension tend to focus on present aspects of living/functioning (“problems with washing or dressing”, “problems doing usual- work, study, housework, family or leisure- activities”). By comparison, the clinical dimension General functioning assesses broader aspects of capability (e.g., “I am not accomplishing as much as I usually do”). Especially, items such as “I function well in the areas that are most important to me” and “I get things done” not only evaluate current functioning but also imply confidence in one’s ability to maintain performance and achieve future goals. Moreover, Hopelessness dimension explicitly addresses future outlook through items like “I have given up hope for a better future” and “No matter how hard I try, things do not get better.” This underlying temporal factor may explain their predictive power beyond EQ-5D-5 L alone.

This temporal orientation aligns with existing literature showing that outcome expectancies (e.g., optimism) and future-oriented cognitive processes (e.g., hope) significantly influence subjective well-being. Scheier and Carver [37] demonstrated that generalized outcome expectancies, particularly optimism about future events, influence health behaviors, while Hirsch et al. [38] found that hope mediated the relationship between functional impairment and depressive symptoms (higher hope in older adults was associated with fewer depressive symptoms despite objective functional limitations). The well-being of individuals with mental health conditions appears to be shaped by factors that extend beyond their current symptoms. By accounting for these deeper, more dynamic consideration, we may be able to bridge the conceptual gap that exists between standard measurement tools and the patient’s actual lived experience.

Moreover, our study provided noteworthy insights into how the EQ-5D-5L structure may function differently within this clinical population compared to the general population. In the general population, dimensions such as ‘Pain/discomfort’ and ‘Mobility’ typically represent distinct issues [32, 39]. However, in our analysis, they loaded onto a single ‘Pain & physical health’ factor, suggesting they represent a closely intertwined experience of bodily distress for individuals with mental disorders. Similarly, EQ-5D-5L ‘Self-care’ and ‘Usual activities’ merged into one ‘Functioning & self-management’ factor. This pattern likely reflects the global functional impairment characteristic of mental health conditions, where patients may consistently experience difficulties in both domains when unwell and improvements in both when recovering, rather than experiencing selective impairments. These results suggest that the five dimension structure of the EQ-5D-5 L may not fully capture the more interconnected and less differentiated nature of suffering as it is subjectively experienced by individuals in specialized mental health treatment.

Our correlation analysis revealed that several clinical dimensions such as Anger, Substance use, Restrictive eating, and Sexuality, showed weak correlations (< 0.30) with all EQ-5D-5 L dimensions. This suggests that these specific clinical constructs either represent dimensions of patient experience that are not captured by the EQ-5D-5 L, or they are constructs whose direct link to HRQoL - as defined by the EQ-5D - is limited. This pattern highlights areas where the EQ-5D-5 L’s comprehensiveness is challenged in a mental health context, impacting how improvements or deteriorations in these symptoms are reflected in HRQoL scores. The weak correlation may also stem from the heterogeneity of our mental health sample, where these symptoms may be profoundly important aspects of disorders for specific sub-populations (e.g., substance use disorders or eating disorders), but completely unimportant for others, thereby diluting the overall correlation. Therefore, supplementary assessment measures that specifically target these constructs should be considered when evaluating therapeutic interventions, as the EQ-5D-5 L alone necessitates the use of additional dimensions for a comprehensive understanding of patient experience. Ultimately, future research should determine whether these domains should be considered essential to the understanding of HRQoL in mental health patients.

A major strength of this study is its clinical applicability. By directly comparing EQ-5D-5 L with 28 clinically relevant dimensions, our findings offer immediately applicable insights for individuals with specific mental health concerns. Our findings identify clinical and socioeconomic dimensions potentially underrepresented by EQ-5D-5 L, highlighting the value of supplementary measures. The mental health dimensions that could complement EQ-5D-5 L were those capturing the active process of psychological suffering - like Internal avoidance, Intrusive memories, Self-contempt, and Hopelessness - and broader domains entirely missed by the generic instrument, such as social functioning, life stability, and behavioral problems.

Several limitations should be noted.

First, a key aspect of this study’s methodological framework is its reliance on outcome measures (EQ-VAS and LS) that were administered as part of the same instruments (EQ-5D-5 L and NF, respectively). This approach was well-suited for our primary, exploratory goal of investigating the internal relationships within tools used for routine clinical care. However, this design may artificially inflate the strength of relationships due to the items having similar presentation. Therefore, the findings should be tested with other measures in future research. A more robust test would involve validating our findings against a fully independent, external benchmark. Second, the SMS-based recruitment resulted in a low response rate of 5.3%, as text messages are often overlooked by recipients. This low rate could suggest the potential for selection bias, which may affect the generalizability of the findings.

## Conclusion

While EQ-5D-5L remains valuable for health economic evaluations and cross-condition comparisons, our results suggest that a more comprehensive assessment including mental health dimensions could provide considerably better understanding of subjective wellbeing in individuals with mental health conditions. This improvement stems at least partly from capturing dimensions entirely missed by the EQ-5D-5L, such as social functioning, life stability, behavioral problems and externalization. This data suggests expanding the single EQ-5D-5L ‘Anxiety/depression’ dimension into a more complete ‘Psychological distress’ factor would be highly beneficial. Rather than simply reporting symptom severity, a more comprehensive assessment of distress includes the active and exhausting process that occurs when individuals struggle against internal pain. Most importantly, it incorporates how patients’ hope and expectations for the future shape their present sense of well-being. These findings can inform the development of more comprehensive assessment tools for mental health populations, improving healthcare decision-making and patient outcomes.
