# Correction: Moving beyond global scores: the added value of clinical dimensions for assessing HRQoL in mental disorders

**DOI:** 10.1007/s11136-026-04258-0

**Published:** 2026-05-14

**Authors:** Yeujin Ki, Andrew Athan McAleavey, Tron Anders Moger, Christian Moltu

**Affiliations:** 1 Department of Research and Innovation, Helse Førde, Svanehaugvegen 2, 6812 Førde, Norway; 2 Department of Psychiatry, Helse Førde, Svanehaugvegen 2, 6812 Førde, Norway; 3 Department of Health and Caring Sciences, Western Norway University of Applied Science, Svanehaugvegen 1, 6812 Førde, Norway; 4 Department of Health Management and Health Economics, University of Oslo, Problemveien 11, 0313 Oslo, Norway

**Correction to: Quality of Life Research (2026) 35:34** 10.1007/s11136-025-04099-3

In Table 2 of this article, entry 0.148 in the fourth column were incorrect and should have been 0.411. The incorrect and corrected version of Table 2 are provided in this correction.

Incorrect version of Table 2:

**Table:** **Table 2**. Regression results predicting EQ-VAS and LS scores

	EQ-VAS		LS	
	Coefficient	*p* value	Coefficient	*p* value
Baseline EQ-5D-5L model				
Mobility	− 0.04	0.54	0.04	0.52
Self-care	0.03	0.61	− 0.01	0.90
Usual activities	− 0.29	< 0.01*	− 0.23	< 0.01*
Pain/discomfort	− 0.12	0.05	− 0.09	0.07
Anxiety/depression	− 0.22	< 0.01*	− 0.50	< 0.01*
Adjusted R^2^	0.235		0.148	
AIC	797.1		716.5	
Comprehensive (EQ-5D-5L + NF) model				
Factor 1: Psychological distress	− 0.36	< 0.01*	− 0.52	< 0.01*
Factor 2: Pain/physical health	− 0.22	< 0.01*	− 0.09	0.04*
Factor 3: Functioning/self-management	− 0.24	< 0.01*	− 0.34	< 0.01*
Factor 4: Behavioral problems/externalizing	0.21	< 0.01*	0.23	< 0.01*
Factor 5: Social/life stability	− 0.03	0.67	− 0.15	< 0.01*
Adjusted R^2^	0.319		0.584	
AIC	761.3		610.0	

Correct version of Table 2:

**Table:** **Table 2**. Regression results predicting EQ-VAS and LS scores

	EQ-VAS		LS	
	Coefficient	*p* value	Coefficient	*p* value
Baseline EQ-5D-5L model				
Mobility	− 0.04	0.54	0.04	0.52
Self-care	0.03	0.61	− 0.01	0.90
Usual activities	− 0.29	< 0.01*	− 0.23	< 0.01*
Pain/discomfort	− 0.12	0.05	− 0.09	0.07
Anxiety/depression	− 0.22	< 0.01*	− 0.50	< 0.01*
Adjusted R^2^	0.235		0.411	
AIC	797.1		716.5	
Comprehensive (EQ-5D-5L + NF) model				
Factor 1: Psychological distress	− 0.36	< 0.01*	− 0.52	< 0.01*
Factor 2: Pain/physical health	− 0.22	< 0.01*	− 0.09	0.04*
Factor 3: Functioning/self-management	− 0.24	< 0.01*	− 0.34	< 0.01*
Factor 4: Behavioral problems/externalizing	0.21	< 0.01*	0.23	< 0.01*
Factor 5: Social/life stability	− 0.03	0.67	− 0.15	< 0.01*
Adjusted R^2^	0.319		0.584	
AIC	761.3		610.0	

The original article has been corrected.

